# Incidental Heterotaxy Syndrome With Polysplenia and Inferior Vena Cava Agenesis Identified During Trauma Evaluation

**DOI:** 10.7759/cureus.104094

**Published:** 2026-02-23

**Authors:** Zainulabideen Ahmed, Shamma Abdallah, Hend Alahmed, Fatemeh Akbarpoor, Labib Al Ozaibi

**Affiliations:** 1 College of Medicine, Mohammed Bin Rashid University of Medicine and Health Sciences, Dubai, ARE; 2 General Surgery, Dubai Academic Health Corporation, Dubai, ARE

**Keywords:** azygous continuation, congenital venous anomaly, deep vein thrombosis risk, hemiazygos continuation, hetrotaxy syndrome, incidental finding, inferior vena cava agenesis, intestinal malrotation, polysplenia, trauma imaging

## Abstract

This case report describes a young adult male in his 20s who was admitted following a high-speed motor vehicle collision resulting in polytrauma, including a femoral shaft fracture. Initial computed tomography performed for polytrauma incidentally revealed heterotaxy syndrome with polysplenia; agenesis of the inferior vena cava with hemiazygos continuation; and intestinal malrotation. Orthopedic management proceeded uneventfully.

This case highlights several important learning points: inferior vena cava agenesis is associated with an increased risk of venous thromboembolism, necessitating heightened awareness and appropriate thromboprophylaxis in trauma and perioperative settings; associated venous anomalies should be clearly documented to inform future procedural planning and patient safety; and asymptomatic intestinal malrotation identified in adulthood can often be managed conservatively with surveillance rather than intervention.

## Introduction

Agenesis of the inferior vena cava (IVC), particularly involving the hepatic segment with azygos or hemiazygos continuation, is a rare congenital vascular anomaly with an incidence of 0.5-1% [1]. It has been described as an incidental finding, often associated with venous thromboembolic disease and complex systemic venous anatomy, in case reports and small series [2]. Although many individuals remain asymptomatic, inferior vena cava agenesis is a recognized predisposing factor for early-onset venous thrombosis, with affected patients typically presenting at a young age (median 34.6 years) and most thrombotic events being unprovoked (≈71%) [3].

Polysplenia represents a variant of heterotaxy characterized by multiple splenic nodules, abnormal systemic venous anatomy, and variable visceral malposition. Adult presentations are increasingly recognized with the widespread use of cross-sectional imaging and are frequently incidental findings during evaluation for unrelated conditions [4], as described in case reports and focused reviews of the adult heterotaxy/polysplenia spectrum. Intestinal malrotation, reported in up to 70% of individuals with heterotaxy syndrome [5], is classically diagnosed in infancy; however, it may also be identified incidentally in adults, who frequently remain asymptomatic yet retain a lifelong risk of volvulus or intestinal obstruction [6].

Recognition of associated vascular anomalies, particularly absent or anomalous superior vena cava (SVC) variants, is essential because these findings may complicate central venous access and cardiothoracic procedures [7].

This case illustrates the incidental discovery of complex congenital anomalies during trauma evaluation and emphasizes their relevance to acute management, perioperative decision-making, and long-term care.

## Case presentation

A previously healthy man in his 20s with no known drug allergies was admitted following a high-speed motor vehicle collision. He reported direct abdominal impact against the steering wheel with prolonged extrication. On arrival, he was conscious, oriented, and not in visible distress. Vital signs were stable, with a blood pressure of 140/85 mmHg, heart rate of 80 beats per minute, respiratory rate of 18 breaths per minute, temperature of 36.8 °C, and oxygen saturation of 99% on room air.

Primary survey revealed a patent airway without cervical spine tenderness, normal breathing, and stable circulation. A deformity of the left lower limb was noted. Neurological examination demonstrated a Glasgow Coma Scale score of 15/15. During the secondary survey, a closed deformity of the left femoral shaft and swelling with effusion of the left knee were identified. The knee was evaluated by the orthopedic team and deemed stable. A 2 cm laceration over the mid-shin extending to bone was irrigated and sutured in the emergency department. The distal neurovascular status of both lower limbs was intact. The right lower limb demonstrated superficial abrasions only. Abdominal examination revealed suprapubic ecchymosis with localized tenderness. Both upper limbs were unremarkable.

**Figure 1:**
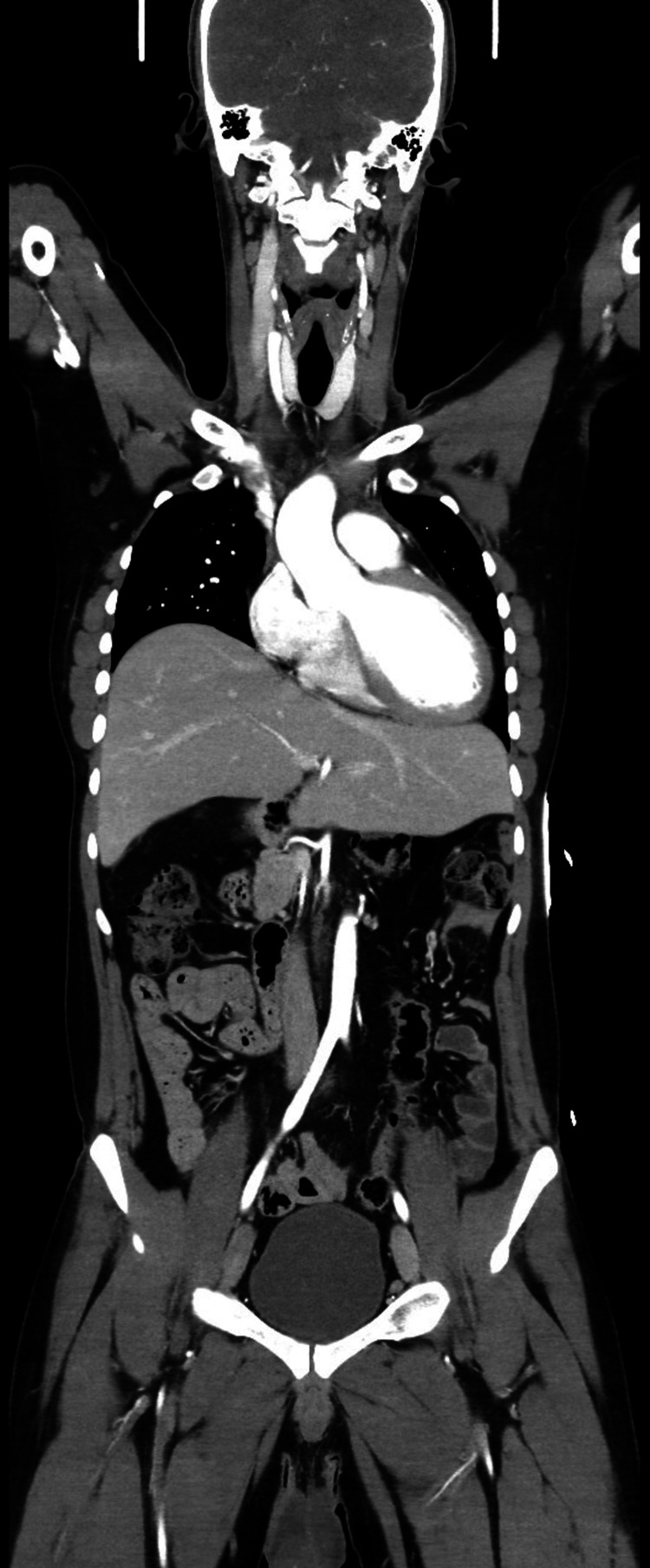
Contrast-enhanced CT demonstrating no evidence of acute traumatic injury to the brain, skull, spine, thorax, pelvis, or solid organs in the abdomen. No free fluid, hemoperitoneum, pneumothorax, or visceral laceration is identified.

As part of the initial polytrauma assessment on admission, a contrast-enhanced CT scan of the head, cervical spine, chest, abdomen, and pelvis was performed. No acute traumatic injury to the brain, spine, thorax, pelvis, or solid organs in the abdomen was identified. There was no free fluid, hemoperitoneum, pneumothorax, or visceral laceration (Figure 1).

**Figure 2:**
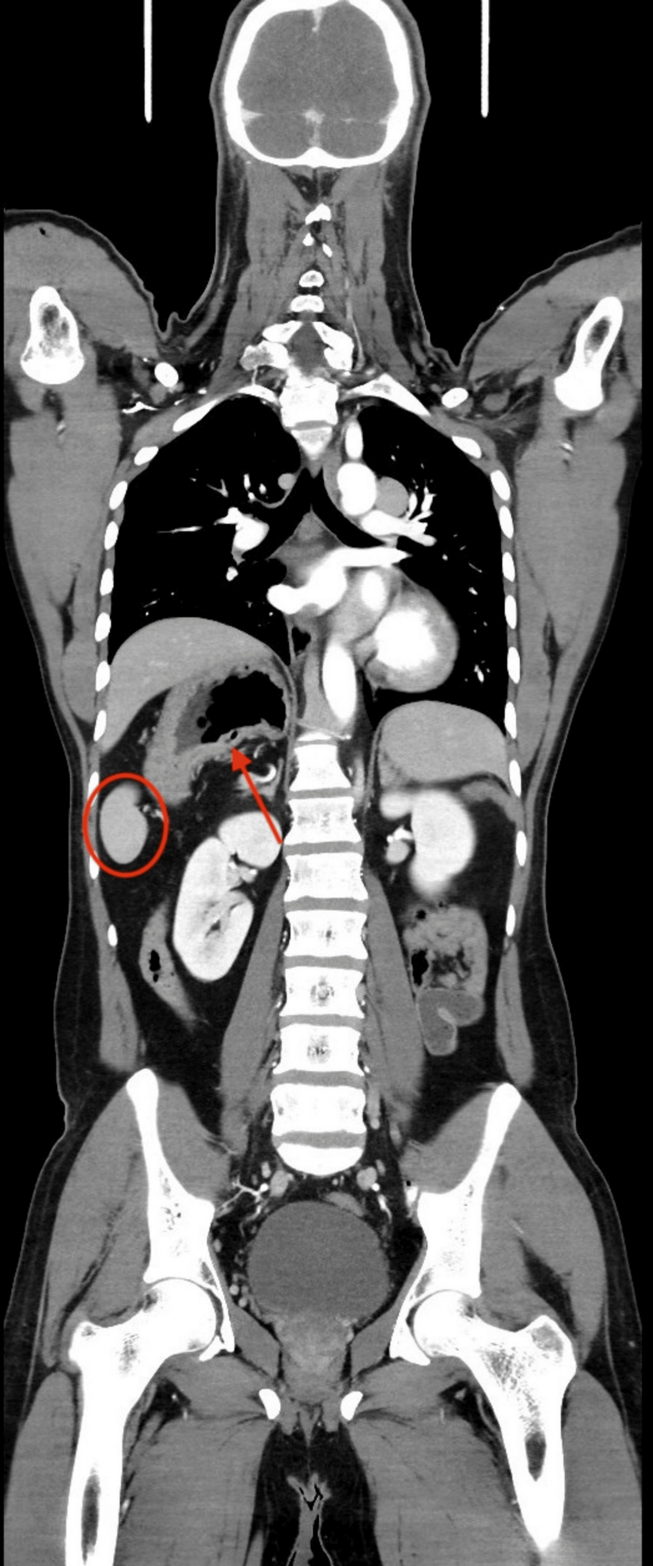
The red arrow indicates a right-sided gastric fundus, consistent with abnormal visceral situs. The red circle highlights right-sided splenic tissue, rather than its normal location in the left upper quadrant.

**Figure 3:**
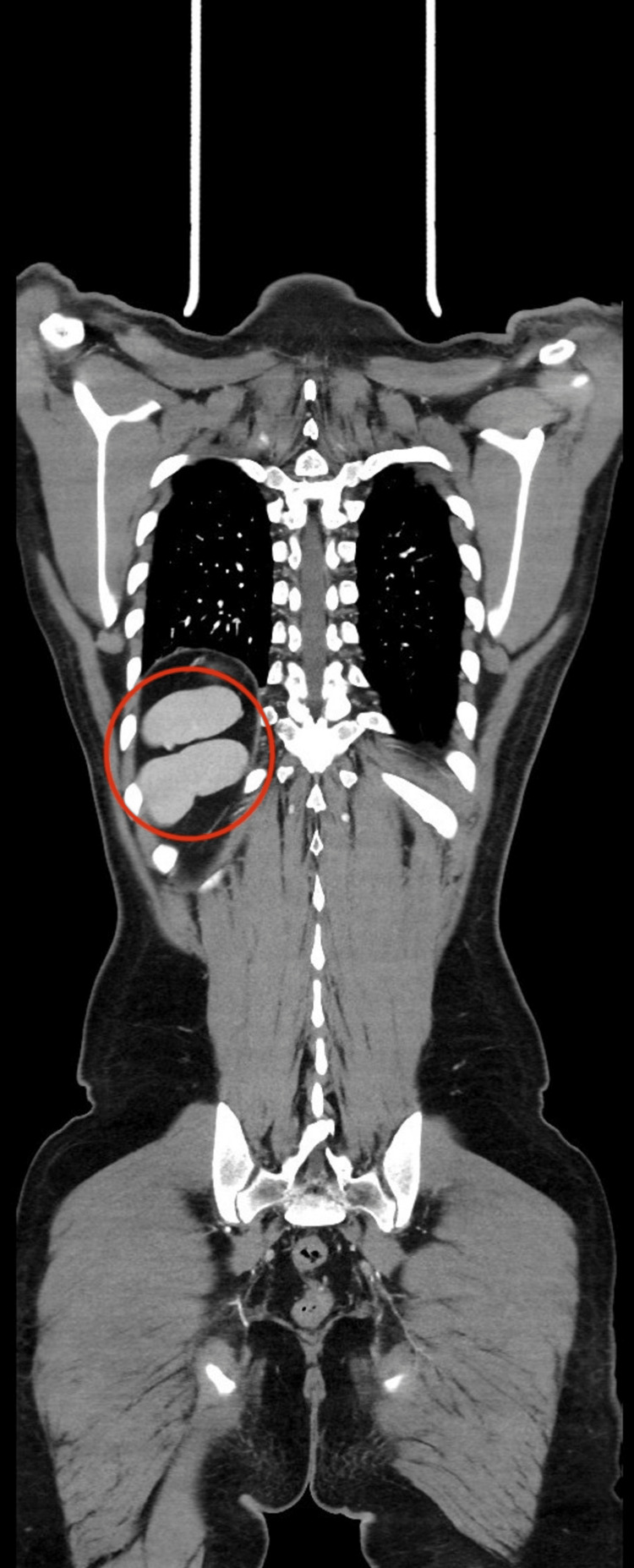
The red circle demonstrates polysplenia, with multiple splenic nodules located in the right subdiaphragmatic region instead of a single spleen in the left upper quadrant.

**Figure 4:**
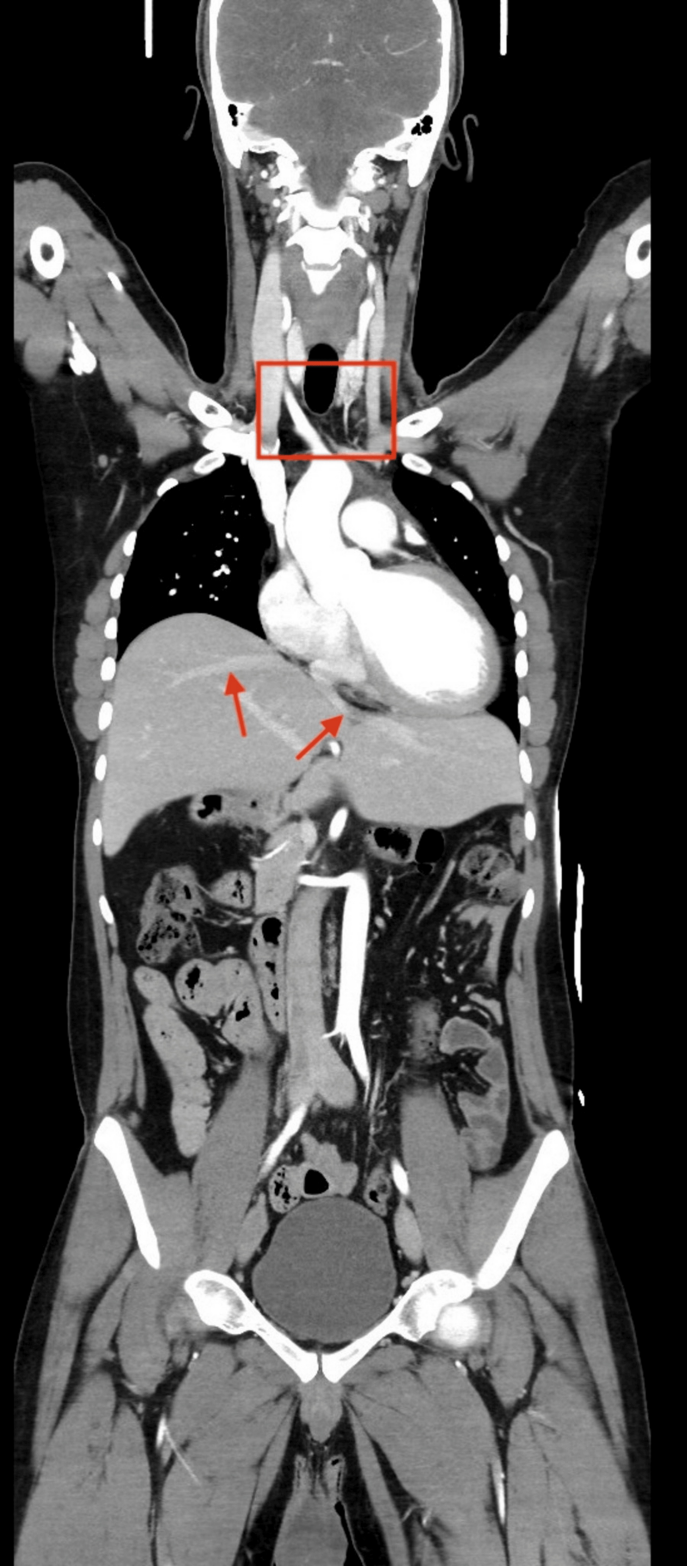
The red arrows demonstrate the right and left hepatic veins draining directly into the right atrium, with the absence of the hepatic segment of the inferior vena cava. The red rectangle highlights the interruption of the suprarenal inferior vena cava with continuation via the azygos-hemiazygos system. The enlarged midline liver extending across both sides of the abdomen is also visible.

**Figure 5:**
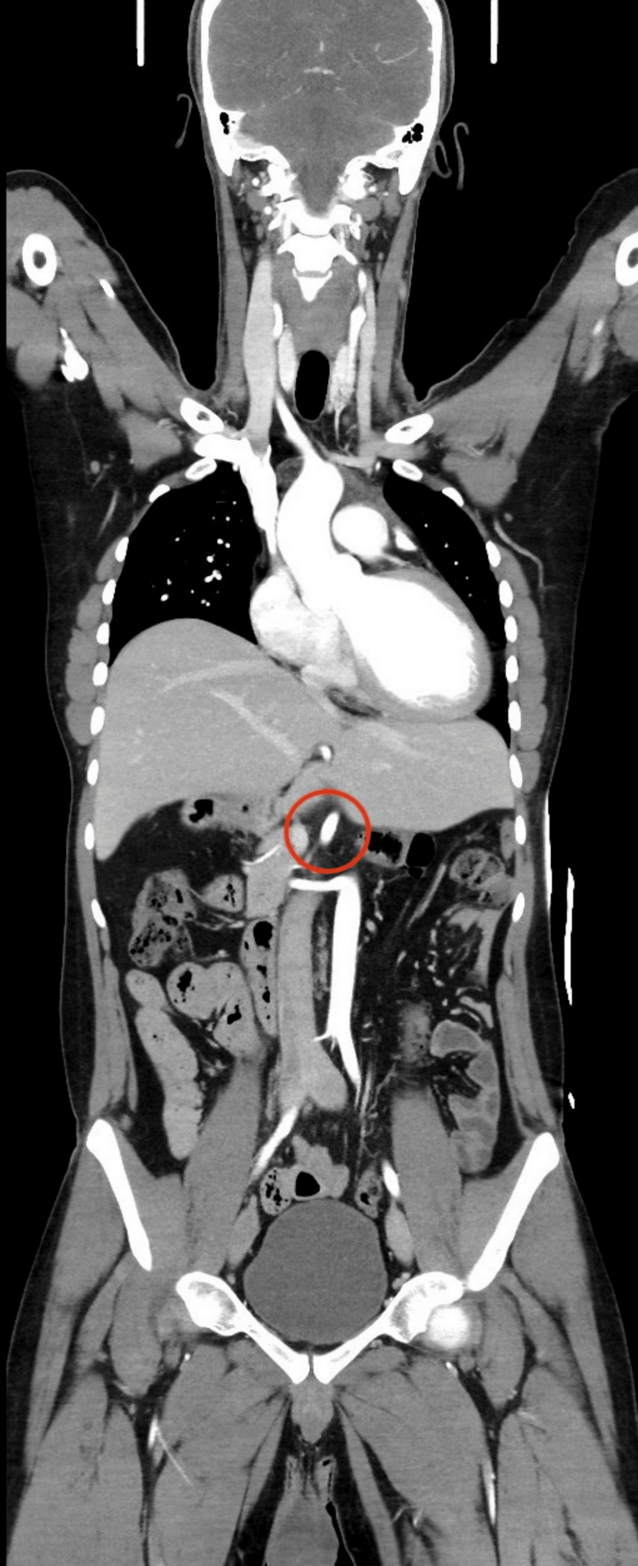
The red circle demonstrates interruption of the suprarenal inferior vena cava, a characteristic vascular anomaly associated with polysplenia syndrome.

Incidental findings consistent with heterotaxy syndrome were identified. These included a right-sided stomach and duodenojejunal flexure with features of intestinal malrotation, polysplenia, and an enlarged midline liver extending across both sides of the abdomen without intrahepatic biliary dilatation. Venous anomalies were notable for the absence of the suprarenal inferior vena cava with direct hepatic venous drainage into the right atrium, the absence of the infrarenal inferior vena cava with hemiazygos continuation, the non-visualization of the superior vena cava, and anomalous renal venous drainage into the hemiazygos system. The portal venous system demonstrated splenic vein-superior mesenteric vein confluence below the level of the renal veins. These anatomical variations are illustrated in Figures 2-5 and detailed in Table 1.

**Table 1:** Congenital anomalies identified on CT imaging Abbreviations: IVC, inferior vena cava; SVC, superior vena cava; SMV, superior mesenteric vein.

System	Specific findings
Venous system	Absence of the suprarenal inferior vena cava (IVC), with the hepatic veins draining directly into the right atrium. Absence of the infrarenal IVC with hypertrophied hemiazygos continuation coursing in the posterior mediastinum. The superior vena cava (SVC) is not visualized. The right brachiocephalic vein drains directly into the right atrium. The left renal vein drains into the hemiazygos system.
Abdominal situs	Intestinal malrotation with a medially positioned ascending colon beneath the transverse colon. The small bowel is predominantly right-sided, with crowding in the right iliac fossa. The stomach and duodenojejunal flexure are located on the right. The descending colon is intraperitoneal. The appendix is not clearly identified.
Hepatobiliary system	The midline, enlarged liver extending across both hemiabdomens is consistent with abnormal visceral lateralization. No intrahepatic biliary dilatation is observed. The portal vein and hepatic veins are patent.
Splenic system	Polysplenia with multiple splenules in the right subdiaphragmatic region, including one adjacent to the gastric fundus, demonstrating heterogeneous enhancement with a small non-enhancing area (possible contusion, infarct, or normal variant).
Pancreas	Distorted pancreatic configuration is positioned on the right side of the abdomen.
Portal venous system	The splenic vein joins the superior mesenteric vein (SMV) below the level of the renal veins to form the portal vein.

On hospital day 1, 4 kg skin traction was applied to the left lower limb, and the patient was kept nil per os in preparation for surgery. He was admitted under the trauma service. On hospital day 2, definitive orthopedic management was performed, consisting of femoral reconstruction nailing, debridement of the mid-shin wound, and open posterior cruciate ligament repair. Postoperative care included intravenous cefazolin for 24 hours, thromboprophylaxis with enoxaparin 40 mg daily, multimodal analgesia, pantoprazole, and strict non-weight-bearing mobilization.

On hospital day 3, the patient developed new-onset abdominal pain localized to the left lower quadrant, associated with obstipation. He was febrile (38.9 °C), hypotensive (77/42 mmHg), and tachycardic (110 beats per minute), raising concern for an acute intra-abdominal process. Abdominal examination revealed a soft, non-distended abdomen with suprapubic and right lower quadrant tenderness, without peritoneal signs. Laboratory findings are summarized in Table 2.

**Table 2:** Results of laboratory investigations on admission and hospital day 4 Initial laboratory studies demonstrated leukocytosis on admission, which normalized by hospital day 4. A mild decline in hemoglobin and hematocrit was observed during hospitalization. Electrolytes and renal function parameters remained within normal limits. Abbreviations: eGFR, estimated glomerular filtration rate; CKD-EPI, Chronic Kidney Disease Epidemiology Collaboration.

Laboratory Test	Units	Result (Admission Day)	Result (Hospital Day 4)	Reference Range
Hematology				
White blood cell count (automated)	10³/µL	14.1	7.0	4.0–11.0
Hemoglobin	g/dL	12.6	11.0	13.5–17.5
Hematocrit	%	37.2	32.0	41–53
Urea and electrolytes				
Sodium	mmol/L	140	-	136–145 mmol/L
Potassium	mmol/L	3.4	-	3.4–4.5 mmol/L
Chloride	mmol/L	105	-	98–108 mmol/L
Bicarbonate	mmol/L	22.1	-	20–28 mmol/L
Urea	mg/dL	27	-	12–40 mg/dL
Anion gap	mmol/L	13	-	6–14 mmol/L
Renal function tests				
Creatinine	mg/dL	0.65 (L)	-	0.70–1.20 mg/dL
eGFR (CKD-EPI)	mL/min/1.73 m²	134.9	-	>60 mL/min/1.73 m²

Cross-sectional imaging demonstrated no evidence of obstruction, perforation, or bowel ischemia. The episode was therefore managed conservatively with intravenous fluids, empiric antibiotics, serial abdominal examinations, and repeat laboratory monitoring. The patient improved clinically with resolution of fever and stabilization of vital signs, supporting a transient inflammatory or reactive process rather than a surgical emergency. No operative intervention was required.

Given the incidental diagnosis of heterotaxy syndrome with polysplenia and intestinal malrotation, the general surgery team recommended outpatient follow-up and counseling regarding potential long-term risks, including midgut volvulus or bowel obstruction.

## Discussion

In the present case, heterotaxy syndrome with polysplenia, bilateral (midline) hepatic morphology, and complex systemic venous anomalies was identified incidentally in an adult trauma patient during cross-sectional imaging performed for acute post-traumatic abdominal symptoms. Heterotaxy syndrome is a rare congenital disorder, with an estimated incidence of approximately 1 in 250,000 live births [8]. It arises from the disruption of normal left-right axis determination during early embryogenesis, a process governed by nodal ciliary function and asymmetric expression of signaling pathways such as NODAL and LEFTY. Failure of this coordinated laterality patterning may result in visceral malposition, splenic abnormalities, and systemic venous anomalies. In polysplenia (left atrial isomerism), the liver often demonstrates a symmetric or midline configuration extending across both hypochondria, reflecting impaired lateralization rather than true hepatomegaly [9]. Agenesis of the inferior vena cava with azygos or hemiazygos continuation is a recognized but uncommon associated vascular abnormality, reported in up to 0.6% of the population, and is often clinically silent outside of thromboembolic complications [2,10].

The principal clinical relevance of inferior vena cava agenesis lies in its well-documented association with venous thromboembolism. Although many congenital IVC anomalies are asymptomatic, clinical series and reviews have highlighted that thromboembolic complications, particularly lower-limb deep vein thrombosis in young adults, can occur in the setting of IVC absence or atresia [10]. The proposed mechanism relates to venous stasis and reliance on collateral pathways, such as the azygos and hemiazygos systems, which may be insufficient during periods of increased venous return or immobilization [3,10]. In the context of major trauma, orthopedic surgery, and prolonged non-weight-bearing status, this underlying anatomy confers additional thrombotic risk. The use of postoperative pharmacologic prophylaxis with low-molecular-weight heparin in this patient is therefore consistent with available evidence and represents an appropriate risk-mitigating strategy.

There are currently no consensus guidelines addressing long-term anticoagulation in asymptomatic individuals with incidentally discovered IVC agenesis. Management decisions remain individualized and are typically guided by the presence of prior thrombotic events, additional risk factors, and patient-specific considerations. Published case reports and narrative reviews describe variable strategies, ranging from short-term anticoagulation following acute thrombosis to prolonged or lifelong therapy in patients with recurrent or unprovoked events [11]. More recent literature has documented the successful use of direct oral anticoagulants in this population, suggesting a potential alternative to traditional vitamin K antagonists when long-term therapy is indicated, although high-quality comparative data are lacking [12].

Polysplenia, as part of the heterotaxy spectrum, is frequently associated with systemic venous anomalies, abnormal splenic venous drainage, and visceral malposition. Adult presentations are increasingly recognized as incidental findings on computed tomography performed for unrelated indications [4]. Intestinal malrotation, although classically diagnosed in infancy, may remain asymptomatic into adulthood. In the absence of volvulus, bowel obstruction, or chronic gastrointestinal symptoms, conservative management with observation and patient education has been described in adult series, although optimal management remains controversial [6]. Although congenital inferior vena cava anomalies are frequently asymptomatic, they may be identified incidentally and are not necessarily responsible for nonspecific or transient abdominal symptoms [13].

Accurate documentation of anomalous systemic venous return, including absent or abnormal superior vena cava drainage, carries important practical implications. Such anomalies may complicate central venous catheter placement, pacemaker or defibrillator implantation, and cardiothoracic as well as abdominal or retroperitoneal surgical procedures, underscoring the need for clear communication in the medical record [7,14].

This case highlights the value of thorough imaging review in trauma patients and emphasizes the long-term clinical significance of incidentally discovered congenital vascular and visceral anomalies.

## Conclusions

This case highlights the incidental identification of heterotaxy syndrome with polysplenia and inferior vena cava agenesis during trauma imaging in an otherwise asymptomatic adult. Although unrelated to the acute presentation, recognition of this complex venous anatomy had direct implications for perioperative risk assessment and thromboprophylaxis planning. Such anomalies may remain clinically silent for decades, yet confer increased susceptibility to venous thromboembolism and procedural challenges. Careful imaging review and clear documentation of aberrant systemic venous return are therefore essential, particularly in patients undergoing surgery or prolonged immobilization. Incidental discovery in adulthood provides a critical opportunity to anticipate thrombotic risk, guide preventive strategies, and ensure safer long-term care.
